# Abemaciclib-induced lichen planus pigmentosus inversus improving after switching to ribociclib^☆^

**DOI:** 10.1016/j.abd.2023.11.007

**Published:** 2024-08-06

**Authors:** Antoine Communie, Isabelle Valo, Patrick Soulié, Xavier Grimaux

**Affiliations:** a Department of Medical Oncology, Institut de Cancérologie de l’Ouest, Angers, France; b Histology and Cytopathology Laboratory, Institut de Cancérologie de l’Ouest, Angers, France

Dear Editor,

Abemaciclib and ribociclib are Cyclin-Dependent Kinase 4/6 (CDK) inhibitors, targeted therapies recently approved for the treatment of hormone receptor-positive and human epidermal growth factor receptor 2-negative breast cancer in association with hormonal therapy, potentially causing various cutaneous adverse events. Here, we report a case of Lichen Planus Pigmentosus Inversus (LPPi) induced by abemaciclib treatment improving after switching to ribociclib.

A 61-year-old woman, who had been treated with abemaciclib for 5 months with a conceded dose of 100 mg/day due to diarrhea was admitted with an asymptomatic flexural pigmentation of the submammary, cubital and inguinal folds. The patient had a Fitzpatrick II skin type and was only taking letrozole and loperamide as other drugs, introduced simultaneously. She showed gray hyperpigmented lesions with papules and desquamation on the flexural areas (Fig. 1, Fig. 2). There was no mucosal involvement. A punch biopsy on the right thigh showed an acanthotic and ortho-keratotic epidermal layer with intraepidermal pagetoid migration of lymphoid cells, band-like lichenoid lymphocytic infiltrate in the papillary dermis with pigmentary incontinence and melanophage (Fig. 3), which confirmed the diagnosis. Abemaciclib was suspended due to the rapidly extensive lesions and digestive toxicity, and the lichenoid drug reaction started to decay after 15 days. Treatment was then switched to ribociclib with the use of topical betamethasone for about a month. The patient stopped using topical corticoids as the hyperpigmented lesions lessened. She has since been treated with ribociclib without any extension of the remaining lesions.

**Fig. 1:**
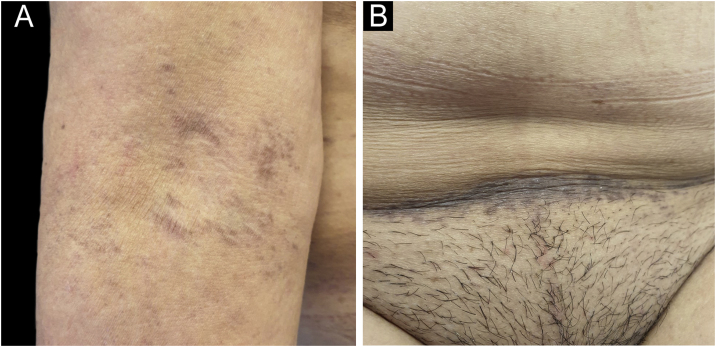
Gray hyperpigmented lesions with papules and desquamation in the cubital (A), and lower abdominal fold (B).

**Fig. 2:**
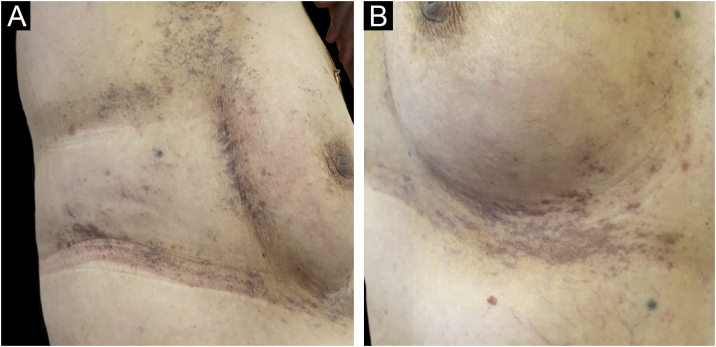
Bilateral submammary reticular gray hyperpigmented lesions with papules and desquamation (A, B).

**Fig. 3:**
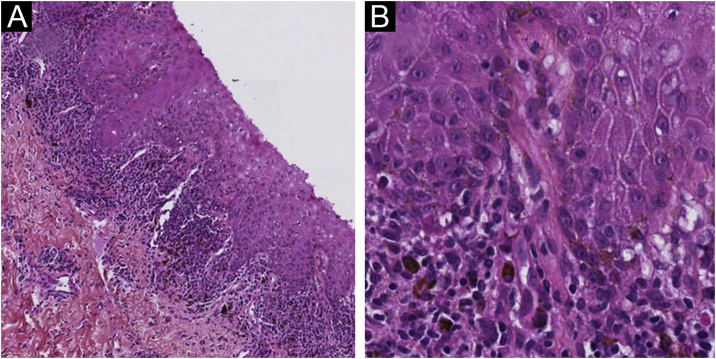
Histopathological findings. Lymphohistiocytic infiltrate with dermal melanin incontinence, lichenoid band-like of the papillary dermis with intraepidermal pagetoid migration and acanthotic epidermal layer. Hematoxylin & eosin ×100 (A) and ×400 (B).

Lichen planus pigmentosus inversus is a rare variant of lichen planus that presents as an acquired symmetrical macular hyperpigmentation featuring gray or blue-brown lesions with erythematous borders in flexural and intertriginous areas, sparing sun-exposed areas. Skin toxicities with CDK4/6 inhibitors consist mostly of alopecia, rash, and pruritus according to a recent review of the US FDA Adverse Event Reporting System by Raschi et al.^1^ To date, only one case of lichen planus pigmentosus has been reported with ribociclib by Mariano et al.,^2^ and one case of hyperpigmentation with abemaciclib by Salusti-Samson et al.^3^ Lichen and lichenoid eruptions have also been described with letrozole, but it was excluded in this case because of the improvement without discontinuation of the treatment. LPPi involves cytotoxic T-lymphocytes, but its pathogenesis remains unknown.

It has been reported in association with mechanical trauma, hepatitis C virus, and after COVID-19 vaccination by Chaima et al.,^4^ which was not the case with our patient. To our knowledge, it is the first reported case of LPPi induced by a CDK4/6 inhibitor that improved after switching to another one. This could be linked to the greater selectivity for CDK4 than CDK6 of abemaciclib versus the others. This suggests that the lichenoid-drug reaction is not a class effect and that a change in the CDK4/6 inhibitor could be a viable option.

## Financial support

None declared.

## Authors’ contributions

Antoine Communie: Approval of the final version of the manuscript; preparation and writing of the manuscript; data collection, analysis, and interpretation; critical literature review; manuscript critical review.

Isabelle Valo: Approval of the final version of the manuscript; preparation and writing of the manuscript; intellectual participation in the propaedeutic and/or therapeutic of the studied case; manuscript critical review.

Patrick Soulié: Approval of the final version of the manuscript; intellectual participation in the propaedeutic and/or therapeutic conduct of the studied case; manuscript critical review.

Xavier Grimaux: Approval of the final version of the manuscript; preparation and writing of the manuscript; collection, analysis, and interpretation of data; intellectual participation in the propaedeutic and/or therapeutic conduct of the studied case; critical literature review; manuscript critical review.

## Conflicts of interest

None declared.
